# A Cell-Based Reporter Assay for Screening Inhibitors of MERS Coronavirus RNA-Dependent RNA Polymerase Activity

**DOI:** 10.3390/jcm9082399

**Published:** 2020-07-27

**Authors:** Jung Sun Min, Geon-Woo Kim, Sunoh Kwon, Young-Hee Jin

**Affiliations:** 1 Herbal Medicine Research Division, Korea Institute of Oriental Medicine, Daejeon 34054, Korea; jsmin1019@kiom.re.kr (J.S.M.); azard3142@kiom.re.kr (G.-W.K.); 2 Center for Convergent Research of Emerging Virus Infection, Korea Research Institute of Chemical Technology, Daejeon 34114, Korea; 3 KM Application Center, Korea Institute of Oriental Medicine, Daegu 41062, Korea

**Keywords:** Middle East respiratory syndrome, coronavirus, RNA-dependent RNA polymerase, cell-based reporter assay, nucleoside analog, remdesivir

## Abstract

Severe acute respiratory syndrome (SARS), Middle East respiratory syndrome (MERS), and coronavirus disease 2019 (COVID-19) are emerging zoonotic diseases caused by coronavirus (CoV) infections. The viral RNA-dependent RNA polymerase (RdRp) has been suggested as a valuable target for antiviral therapeutics because the sequence homology of CoV RdRp is highly conserved. We established a cell-based reporter assay for MERS-CoV RdRp activity to test viral polymerase inhibitors. The cell-based reporter system was composed of the bicistronic reporter construct and the MERS-CoV nsp12 plasmid construct. Among the tested nine viral polymerase inhibitors, ribavirin, sofosbuvir, favipiravir, lamivudine, zidovudine, valacyclovir, vidarabine, dasabuvir, and remdesivir, only remdesivir exhibited a dose-dependent inhibition. Meanwhile, the Z-factor and Z′-factor of this assay for screening inhibitors of MERS-CoV RdRp activity were 0.778 and 0.782, respectively. Ribavirin and favipiravir did not inhibit the MERS-CoV RdRp activity, and non-nucleoside HCV RdRp inhibitor, dasabuvir, partially inhibited MERS-CoV RdRp activity. Taken together, the cell-based reporter assay for MERS-CoV RdRp activity confirmed remdesivir as a direct inhibitor of MERS-CoV RdRp in cells. A cell-based MERS-CoV RdRp activity reporter assay is reliable and accurate for screening MERS-CoV RdRp-specific inhibitors. It may provide a valuable platform for developing antiviral drugs for emerging CoV infections.

## 1. Introduction

Severe acute respiratory syndrome (SARS), Middle East respiratory syndrome (MERS), and coronavirus disease 2019 (COVID-19) are emerging zoonotic diseases caused by SARS coronavirus (SARS-CoV), MERS-CoV, and SARS-CoV-2 infections, respectively. These CoVs belong to the *β-coronavirus* genus, and they can cause severe acute respiratory infection (SARI) with multi-organ failure, resulting in a high fatality rate.

MERS-CoV is transmitted from dromedary camels and/or bats to the human population [1]. Primary infection has typically occurred in Middle Eastern countries; however, the 2015 MERS-CoV outbreak centered on South Korea. This indicated that the emerging viral infection is not predictable, and thus, this virus should not be solely considered a domestic issue. Although MERS-CoV infection has a high mortality rate, no approved therapeutics or vaccines have been developed to date. Therefore, in 2018, the World Health Organization sought to accelerate antiviral research and development of treatments for MERS-CoV [2].

MERS-CoV has a large, positive-sense single-stranded RNA genome of 30.1 kb that expresses both structural and nonstructural proteins. MERS-CoV RNA is inserted into cells via dipeptidyl peptidase 4-mediated endocytosis, and it carries two polyprotein genes (pp1a and pp1ab) that can be transcribed into 16 nonstructural proteins (NSPs) necessary for viral replication and transcription [3]. MERS-CoV polyproteins are self-cleaved into papain-like protease (PLpro) and 3C-like protease (3CLpro), which subsequently cleave the polyproteins to produce the replicative enzymes RNA-dependent RNA polymerase (RdRp), helicase, and exonuclease [4]. Among them, MERS-CoV RdRp is a 106.9-kDa protein encoded by the nsp12 gene that is expressed early in the course of infection and is critical for viral replication [5].

Suppression of MERS-CoV replication by inhibiting MERS-CoV NSPs has been one of the primary antiviral strategies against MERS-CoV infection. Liang et al. recently reviewed several PLpro and 3CLpro inhibitors [6], and the MERS-CoV helicase inhibitor was SSYA10-001, initially developed as a helicase inhibitor of SARS-CoV [7]. However, antivirals targeting MERS-CoV RdRp have not been reported. Nucleoside analogs commonly target viral replication, particularly viral polymerase [8], and they have succeeded clinically in treatment of multiple viral infectious diseases [9]. For example, hepatitis C virus (HCV) RdRp inhibitors have achieved high success rates in clinical treatment [10]. Ribavirin triphosphate inhibits HCV RdRp [11], and sofosbuvir is a nucleotide analog that is inserted into HCV RNA by HCV RdRp, acting as a chain terminator [12]. Dasabuvir is a non-nucleoside HCV NS5B inhibitor that binds to the palm domain of HCV RdRp and induces a conformational change [13]. Therefore, MERS-CoV RdRp inhibition could represent an optimal antiviral strategy.

Ribavirin and remdesivir (GS-5734) are the representative nucleoside analogs that inhibit in vitro MERS-CoV infection [5,14]. To the best of our knowledge, the inhibitory effect of ribavirin on MERS-CoV RdRp activity has not yet been evaluated. The target of remdesivir in treating coronavirus infection was suggested to be the resistance mutation of viral polymerase in mouse hepatitis virus (MHV) and SARS-CoV [5]. It was recently reported that the triphosphate form of remdesivir inhibits MERS-CoV RdRp by using an enzyme activity assay in a cell-free system [15]. However, there is no report describing an antiviral compound that suppresses MERS-CoV RdRp activity in a cell-based reporter assay system.

Lee et al. previously established a cell-based reporter assay system using HCV NS5B and a bicistronic reporter gene, which they used for HCV RdRp inhibitor screening by measuring intracellular HCV RdRp activity [16]. We established a cell-based MERS-CoV RdRp reporter assay system by modifying the previously developed system. Then, we evaluated the inhibitory effect of various viral polymerase inhibitors, including HCV RdRp, negative-strand influenza virus polymerase, human immunodeficiency virus (HIV) reverse transcriptase, and herpes simplex virus (HSV) DNA polymerase, on MERS-CoV RdRp activity and investigated the reproducibility and reliability of this assay for expanded application in high-throughput screening (HTS). We confirmed remdesivir as a direct MERS-CoV RdRp inhibitor using the cell-based assay and it suggested that the newly established cell-based reporter assay is suitable for the rapid and accurate screening of specific MERS-CoV RdRp inhibitors.

## 2. Materials and Methods

### 2.1. Test Compounds

Favipiravir (PubChem CID: 492405), sofosbuvir (PubChem CID: 45375808), and Dasabuvir (PubChem CID: 56640146) were purchased from Chemscene LLC (Monmouth Junction, NJ, USA). Ribavirin (PubChem CID: 37542), valacyclovir hydrochloride (PubChem CID: 135398741), vidarabine monohydrate (PubChem CID: 32326), lamivudine (PubChem CID: 60825), and zidovudine (PubChem CID: 35370) were purchased from Tokyo Chemical Industry CO., Ltd. (Tokyo, Japan). Remdesivir (PubChem CID: 121304016) was purchased from LALPharm Co., Ltd. (Beijing, China). Quality control of remdesivir was guaranteed by the supplier based on HPLC-Mass spec and NMR analysis data, and we re-verified these findings before experimentation. All compounds were stored as 20-mM stock solutions in 100% dimethyl sulfoxide (DMSO) (Sigma-Aldrich, St. Louis, MO, USA).

### 2.2. Plasmids

**Figure 1:**
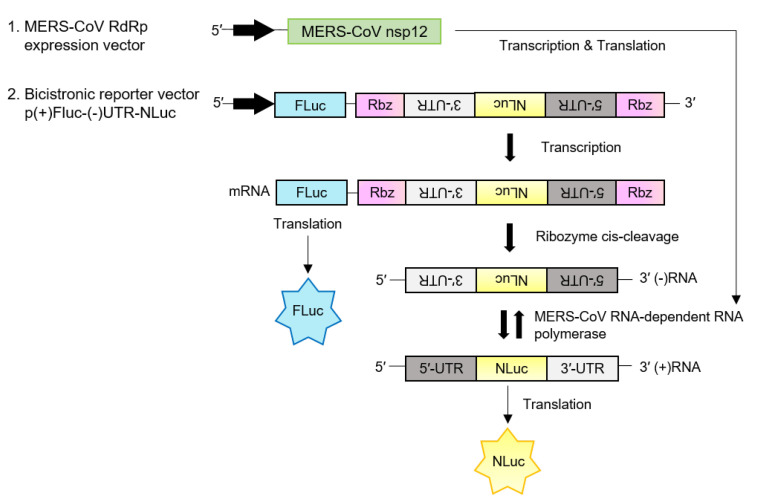
Schematic diagram of the cell-based reporter assay system for Middle East respiratory syndrome coronavirus (MERS-CoV) RNA-dependent RNA polymerase (RdRp) activity. This system is composed of the human codon-optimized FLAG-tagged MERS-CoV nsp12 plasmid construct for MERS-CoV RdRp expression and the bicistronic reporter construct p(+)FLuc-(−)UTR-NLuc, which contains the firefly luciferase gene in the sense orientation, (+)FLuc and the Nano-glo^®^ luciferase gene in the antisense orientation, (−)NLuc, which is flanked by the antisense 5′-untranslated region (UTR) and 3′-UTR of MERS-CoV and the hepatitis delta virus (HDV) ribozyme self-cleavage sequence. The full-length p(+)FLuc-(−)UTR-NLuc RNA transcripts are processed by the HDV ribozyme via self-cleavage, and the exposed (−)NLuc flanked by the antisense 5′- and 3′-UTR RNA can be replicated by MERS-CoV RdRp. Then, the replicated (+)NLuc RNA is translated, and the expression level of NLuc represents the activity of MERS-CoV RdRp. The expression level of FLuc serves as an internal control to normalize NLuc activity.

The N-terminal (N-term) or C-terminal (C-term) Flag-tagged MERS-CoV nsp12 gene, the C-term Flag-tagged nsp7 gene, nsp7 gene, C-term Flag-tagged nsp8 gene, and nsp8 gene (GenBank^®^ accession no. KT029139) were human codon-optimized, synthesized via GENEWIZ (South Plainfield, NJ, USA), and cloned into the *Nhe*I and *Xho*I sites of the pcDNA3.1(+) plasmid (Invitrogen Corporation, Carlsbad, CA, USA) to generate pN-termFlag-nsp12 or pC-termFlag-nsp12 plasmids, pC-termFlag-nsp7 plasmid, pNsp7 plasmid, pC-termFlag-nsp8 plasmid, and pNsp8 plasmid, respectively. To generate the reporter plasmid, we modified the previously published bicistronic HCV RdRp reporter construct [16]. The sense orientation (+) firefly luciferase gene (FLuc) was amplified by PCR from pGL3-basic (Promega Corporation, Madison, WI, USA, GenBank^®^ accession no. U47295) and cloned into the *Nhe*I and *Hind*III sites of the pcDNA3.1(+) plasmid. Subsequently, the hepatitis delta virus (HDV) ribozyme sequence, antisense 3′-untranslated region (UTR) of MERS-CoV, antisense Nano-glo^®^ luciferase gene (NLuc) (Promega Corporation, GenBank^®^ accession no. KM359770), antisense 5′-UTR of MERS-CoV (with/without HCV internal ribosome entry site (IRES) sequence), and HDV ribozyme sequence were synthesized in a row via GENEWIZ and cloned into the *Hind*III and *Xho*I sites downstream of the (+)FLuc gene to generate the p(+)FLuc-(−)UTR-NLuc reporter plasmid (with or without the antisense HCV IRES sequence between (−)5′-UTR and (−)NLuc sequence). p(+)FLuc-(−)UTR-NLuc, the reporter plasmid for the MERS-CoV RdRp activity assay, is presented in Figure 1.

### 2.3. Cells and Transfection

HEK293T cells (30 passages) were obtained from American Type Culture Collection (Manassas, VA, USA). These cells were cultured in Dulbecco’s modified Eagle’ medium (Corning Incorporated, Corning, NY, USA) containing 10% fetal bovine serum (Gibco, Carlsbad, CA, USA) and 1% penicillin/streptomycin (Gibco) at 37 °C in 5% CO_2_. For transient transfection, HEK293T cells were seeded at 96-well plates (Corning) overnight. Plasmids were mixed with TransIT^®^-LT1 transfection reagent (Mirus Bio LLC, Madison, WI, USA) and the plasmid and reagent mixture were added to cells according to the manufacturer’s instructions.

### 2.4. Western Blot Assay

HEK293T cells were seeded in 24-well plates and transfected with pN-termFlag-nsp12 or pC-termFlag-nsp12 plasmids. After 24 h, cells were lysed in Glo Lysis Buffer (Promega Corporation). Lysates were separated via 8% sodium dodecyl sulfate–polyacrylamide gel electrophoresis and transferred to nitrocellulose membranes (Bio-Rad Laboratories, Hercules, CA, USA). The membranes were blocked with 5% skim milk in phosphate-buffered saline with Tween 20 (PBST) for 30 min at room temperature, rinsed with PBST, and incubated with antibodies against FLAG (Cat no. ab125243, Lot no. GR3205413-1, Abcam plc, Cambridge, UK) or β-actin (Cat no. 3700S, Lot no. 15, Cell Signaling Technology, Inc., Danvers, MA, USA) at 4 °C overnight. After three washes with PBST, membranes were incubated with HRP-conjugated secondary antibodies (Cat no. ab6728, Lot no. GR3200472-2, Abcam plc) for 1 h, and detection was performed with Enhanced Chemiluminescence Western Blotting Substrate (Thermo Fisher Scientific, Waltham, MA, USA) using the ChemiDoc™ Touch Imaging System (Bio-Rad Laboratories).

### 2.5. Cytotoxicity Assay

HEK293T cells were seeded into 96-well plates (Thermo Fisher Scientific, Waltham, MA, USA) overnight and then treated with the indicated compounds for 18 h. Cytotoxicity was measured using the CellTiter 96^®^ AQueous One Solution Cell Proliferation Assay (Promega Corporation) according to the manufacturer’s instructions. The absorbance was measured at a wavelength of 490 nm using a GloMax^®^ Discover Microplate Reader (Promega Corporation).

### 2.6. Cell-Based MERS-CoV RdRp Activity Assay

HEK293T cells were seeded in 96-well plates overnight and transfected with pN-termFlag-nsp12 and p(+)FLuc-(−)UTR-NLuc reporter plasmids for 24 h. Cells were treated with the test compounds or 0.25% DMSO (control), starting 6 h after transfection. FLuc and NLuc reporter gene expression in these cells was measured using a Nano-Glo^®^ Dual-Luciferase^®^ Reporter Assay System (Promega Corporation) following the manufacturer’s instructions. The relative activity of MERS-CoV RdRp was determined by normalizing the level of NLuc activity to that of FLuc (NLuc/FLuc ratio). The half-maximal inhibitory concentration (IC_50_), which denoted the concentration at which NLuc activity was reduced by 50% compared with the control level, was measured using linear interpolation.

### 2.7. Calculation of Z-Factor and Z′-Factor

To assess the reliability and reproducibility of the developed assay, Z-factor and Z′-factor values were evaluated using the method of Zhang et al. [17]. First, we executed the assay as mentioned previously, and the experimental grouping was as follows: (1) negative group (n = 40 wells), treated with 0.025% DMSO following the dual transfection of p(+)FLuc-(−)UTR-NLuc and pcDNA3.1 (control vector); (2) positive group (n = 40 wells), treated with 0.025% DMSO following the dual transfection of p(+)FLuc-(−)UTR-NLuc and pN-termFlag-nsp12; and (3) inhibitor group (n = 40 wells); treated with 12 μM remdesivir following the dual transfection of p(+)FLuc-(−)UTR-NLuc and pN-termFlag-nsp12. Z-factor was calculated using the following equation: Z-factor = 1 − [(3SD_Negative_ + 3SD_Positive_)/|mean_Negative_ − mean_Positive_|]. Z′-factor was calculated as follows: Z′-factor = 1 − [(3SD_Inhibitor_ + 3SD_Positive_)/|mean_Inhibitor_ − mean_Positive_|]. For both formulae, the standard deviation and mean values of each group correspond to the relative NLuc activity obtained from each group.

### 2.8. Statistical Analysis

The data were presented as the mean ± SEM. Statistical comparison of luciferase activities by two-way analysis of variance (ANOVA) followed by Bonferroni’s multiple comparison’s test and two-tailed Student’s *t*-test, and non-linear regression analysis of IC_50_ and CC_50_ were conducted using GraphPad Prism^®^ Software V.6.05 for Windows (GraphPad Software Inc., San Diego, CA, USA). *p* values of less than 0.05 were indicated statistically significant.

## 3. Results

### 3.1. Generation of the Cell-Based MERS-CoV RdRp Activity Reporter Assay System

To develop the cell-based MERS-CoV RdRp activity reporter assay, we modified the previously reported cell-based HCV RdRp activity assay [16]. The cell-based reporter system used the bicistronic reporter construct p(+)FLuc-(−)UTR-NLuc, which contains the firefly luciferase gene in the sense orientation, (+)FLuc and Nano-glo^®^ luciferase in the antisense orientation, and (−)NLuc, which is flanked by the antisense 3′- and 5′-UTR of MERS-CoV and the hepatitis delta virus (HDV) ribozyme self-cleavage sequence.

The full length of bicistronic (+)FLuc-(−)UTR-NLuc RNA is transcribed by the host cellular DNA-dependent RNA polymerase *Pol* II. The transcripts are processed by HDV ribozyme self-cleavage, and the exposed negative strand of NLuc flanked by the antisense 3′- and 5′-UTR RNA can be replicated by MERS-CoV RdRp. Then, the replicated positive strand of NLuc RNA is translated, and the expression level of NLuc represents the activity of MERS-CoV RdRp. The expression level of FLuc serves as an internal control of transcription/translation to minimize the variation among samples. p(+)FLuc-(−)UTR-NLuc and the concept of the cell-based MERS-CoV RdRp activity assay are presented in Figure 1.

### 3.2. Evaluation of MERS-CoV RdRp Expression and Optimization of the Reporter Assay System

**Figure 2:**
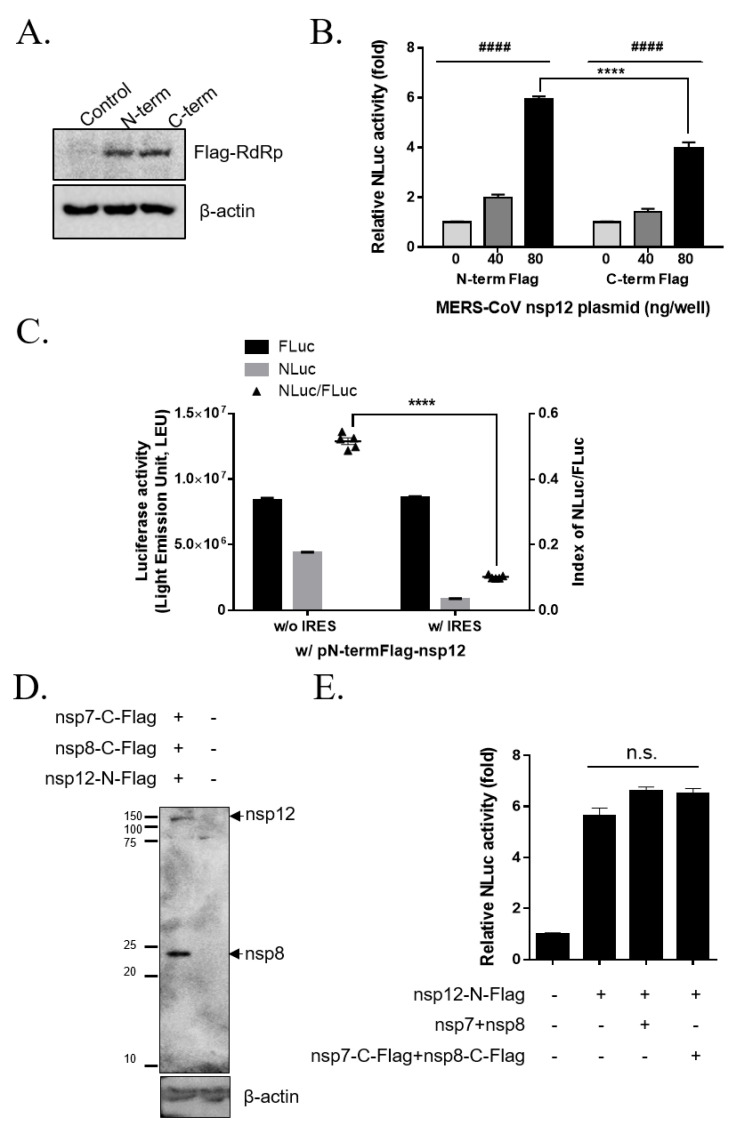
Confirmation of the expression and activity of Middle East respiratory syndrome coronavirus (MERS-CoV) RNA-dependent RNA polymerase (RdRp). (**A**) Expression of the N-terminal (N-term) or C-terminal (C-term) FLAG-tagged MERS-CoV RdRp. HEK293T cells were transfected with pN-termFlag-nsp12 or pC-termFlag-nsp12 plasmids for 24 h. N-term and C-term Flag-tagged RdRp protein was detected as a 110-kDa band via western blotting. β-actin was used as a loading control. (**B**) Comparison of N-term and C-term 110-kDa MERS-CoV RdRp protein activity. HEK293T cells were transfected with 0, 40, or 80 ng of pN-termFlag-nsp12 (N-term) or pC-termFlag-nsp12 (C-term) plasmids together with *p*(+)FLuc-(−)UTR-NLuc. After 24 h, FLuc and NLuc activities were detected using the Nano-glo^®^ Dual-Luciferase^®^ Reporter Assay System, and the relative NLuc activity was normalized by the FLuc activity. (n = 3; flag-tag effect, F_1,12_ = 51.55, *p* < 0.0001; plasmid dose effect, F_2,12_ = 429.7, *p* < 0.0001; flag-tag ⨯ plasmid dose interaction, F_2,12_ = 25.12, *p* < 0.0001; N-term 80 ng vs C-term 80 ng, *p* < 0.0001) (**C**) Comparison of MERS-CoV RdRp activity following transfection of *p*(+)FLuc-(−)UTR-NLuc with or without the antisense hepatitis C virus internal ribosome entry site element plasmid. After transfection with the indicated plasmids and pN-termFlag-nsp12 plasmid into HEK293T cells, FLuc and NLuc activities were measured and the NLuc/FLuc ratio were determined. (n = 5; t_4.426_ = 39.54, *p* < 0.0001) (**D**) Expression of the N-terminal Flag-tagged MERS-CoV RdRp (nsp12-N-Flag) and the C-terminal Flag-tagged nsp8 (nsp8-C-Flag). HEK293T cells were transfected with pN-termFlag-nsp12, pC-termFlag-nsp7, and pC-termFlag-nsp8 plasmids for 24 h. N-term Flag-tagged RdRp protein (nsp12-N-Flag) and C-term Flag-tagged nsp8 (nsp8-C-Flag) were detected as a 110-kDa band and a 22-kDa, respectively via Western blotting. β-actin was used as a loading control. (**E**) Comparison of MERS-CoV RdRp activity following transfection with pC-term Flag-tagged nsp7 (nsp7 C-Flag) and pC-term Flag-tagged nsp8 plasmids (nsp8-C-Flag), or pNsp7 (nsp7) and pNsp8 (nsp8 plasmids. After transfection with the indicated plasmids and *p*(+)FLuc-(−)UTR-NLuc plasmid into HEK293T cells, FLuc and NLuc activities were measured and the NLuc/FLuc ratio were determined. (n = 3; F_3,8_ = 175.3, *p* < 0.0001) Data are presented as the mean ± SEM. Statistical significance was analyzed using one or two-way analysis of variance (ANOVA) followed by Bonferroni’s multiple comparison and two-tailed Student’s *t*-test. #### *p* < 0.0001; **** *p* < 0.0001. The data are representative of at least three independent experiments.

To express MERS-CoV RdRp in a human cell line, we generated the human codon-optimized FLAG-tagged MERS-CoV nsp12 plasmid construct. It was previously reported that the N-term foreign sequence of the poliovirus RdRp affected polymerase activity because the N-term was important for protein folding and the positioning of the active site [18,19]. Therefore, we generated pN-termFlag-nsp12 and pC-termFlag-nsp12 plasmids and compared the expression and activity of MERS-CoV RdRp. We confirmed the expression of N-term or C-term FLAG MERS-CoV RdRp at a molecular weight of approximately 110 kDa following transient transfection with pN-termFlag-nsp12 or pC-termFlag-nsp12 plasmids (Figure 2A).

To compare the activity of N-term or C-term FLAG-tagged MERS-CoV RdRp, luminescence was measured in HEK293T cells co-transfected with p(+)FLuc-(−)UTR-NLuc. The relative NLuc activity was increased by the expression of N-term or FLAG-tagged MERS-CoV RdRp in a dose-dependent manner (Figure 2B), and the luciferase activities were comparable. Therefore, we found that the N-term FLAG tag did not interrupt the activity of MERS-CoV RdRp. Furthermore, the relative NLuc activity of N-term FLAG-tagged MERS-CoV RdRp (5.91 ± 0.14-fold with 80 ng of plasmid) was significantly higher than that of C-term FLAG-tagged MERS-CoV RdRp (3.9 ± 0.26-fold with 80 ng of plasmid). Thus, we used the pN-termFlag-nsp12 plasmid for the cell-based MERS-CoV RdRp activity reporter assay system in the present study.

In the previous cell-based HCV RdRp activity assay, the HCV 5′-UTR comprised an IRES element, which is important for the second cistron luciferase protein translation after HCV NS5B polymerase replicates the positive-strand luciferase RNA [16]. Because we used the MERS-CoV 5′-UTR for the MERS-CoV RdRp reporter assay, we generated p(+)FLuc-(−)UTR-NLuc with or without the antisense HCV IRES element between the (−)5′-UTR and (−)NLuc sequence and then compared the relative NLuc activity of MERS-CoV RdRp. When we transfected p(+)FLuc-(−)UTR-NLuc with or without the antisense HCV IRES element plasmid and pN-termFlag-nsp12 plasmid into HEK293T cells, the relative NLuc activity was compromised by co-transfection of p(+)FLuc-(−)UTR-NLuc and the antisense HCV IRES element plasmid transfection compared with that for p(+)FLuc-(−)UTR-NLuc without the antisense HCV IRES element (Figure 2C). Therefore, we found that the MERS-CoV 5′-UTR does not need the HCV IRES sequence to translate the NLuc protein. 

It was reported that SARS-CoV needs the interacting proteins, nsp8 and nsp7, to have the activity in the enzymatic activity assay because the SARS-CoV nsp8 subunit that forms a hexadecameric complex with nsp7 functions as a RNA primase [20,21]. So, we generated human codon-optimized nsp7 or nsp 8 with or without C-terminal Flag-tagged expression vectors and compared the activity of MERS-CoV RdRp with or without nsp7 and nsp8 protein by determining the relative NLuc activity of MERS-CoV RdRp in this system. After transient transfection with pN-termFlag-nsp12, pC-termFlag-nsp7 and pC-termFlag-nsp8 plasmids, we confirmed the expression of N-term Flag-tagged MERS-CoV RdRp, and the expression of C-term Flag-tagged nsp8 at a molecular weight of approximately 23 kDa (Figure 2D). However, we could not detect the C-term Flag-tagged nsp7 proteins by Western blot analysis in our system. Then, in this cell-based system, the addition of the nsp8 and nsp7 proteins with or without C-terminal Flag tag did not have any significant effect on the relative NLuc activity (Figure 2E). We also suggested the control experiment in cells transfected with the other viral protein, nsp5 plasmid, instead of the nsp12-expressing plasmid, to rule out a possible role of host enzymes in the outcome (Figure S1). Taken together, we finally elected p(+)FLuc-(−)UTR-NLuc, without the antisense HCV IRES element, and pN-termFlag-nsp12, without the addition of nsp7 and nsp8 plasmids, for the cell-based MERS-CoV RdRp activity reporter assay system as the optimized condition.

### 3.3. Effects of Nucleoside/Nucleotide Analogs on MERS-CoV RdRp Activity

To identify nucleoside analogs that could directly target MERS-CoV RdRp activity, drugs including ribavirin, sofosbuvir, favipiravir, lamivudine, zidovudine, valacyclovir, and vidarabine were examined using the optimized assay system.

**Figure 3:**
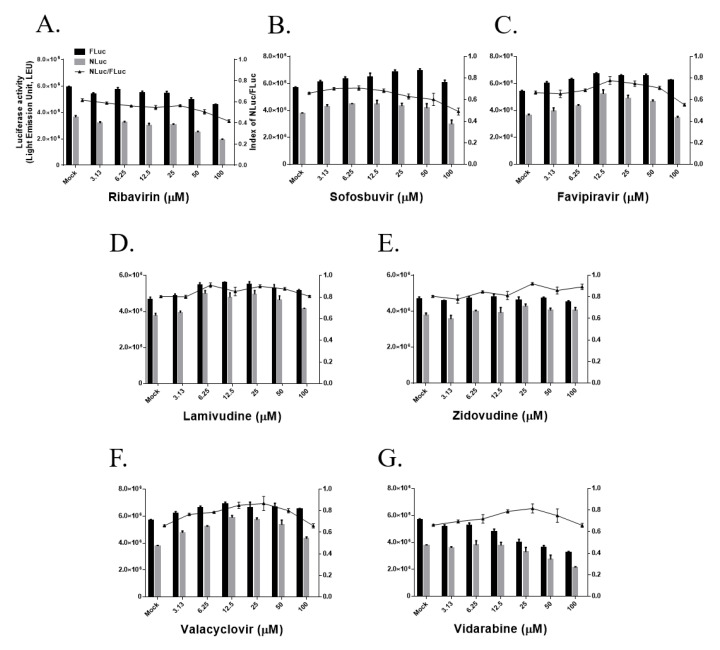
Effects of nucleoside/nucleotide analogs on Middle East respiratory syndrome coronavirus RNA-dependent RNA polymerase (RdRp) activity in the cell-based reporter assay system. HEK293T cells were transiently transfected with p(+)FLuc-(−)UTR-NLuc and pN-termFlag-nsp12, and after 6 h, cells were treated with ribavirin (**A**), sofosbuvir (**B**), favipiravir (**C**), lamivudine (**D**), zidovudine (**E**), valacyclovir (**F**), or vidarabine (**G**) at the indicated concentrations for 18 h. FLuc and NLuc activities were measured and the NLuc/FLuc ratio were determined. The data, which are representative of at least three independent experiments, are presented as the mean ± SEM.

Ribavirin is a guanosine analog and a broad-spectrum antiviral approved for the treatment of RSV [22], HCV [23], Crimean-Congo hemorrhagic fever virus, Lassa virus, and Hantavirus infection based on its ability to prevent viral RNA synthesis [24]. Previous research reported that ribavirin inhibited in vitro MERS-CoV infection in Vero RML6 and LLC-MK2 cells with IC_50_ values of 41.45 and 13.26 μg/ml, respectively [14]. In the present study, 100 μM ribavirin inhibited MERS-CoV RdRp activity by approximately 40% (Figure 3A, Table 1). Sofosbuvir is a clinically approved uridine nucleotide that blocks the HCV NS5B protein, also known as RdRp [25]. However, 100 μM sofosbuvir also only inhibited MERS-CoV RdRp activity by approximately 40% (Figure 3B, Table 1).

Favipiravir (T-705) acts as a purine analog and inhibits the influenza viral polymerase by inducing lethal RNA transversion mutations [26]. Although favipiravir has known to inhibit the RdRp protein in various RNA viruses [27], it only reduced MERS-CoV RdRp activity by approximately 10% at a concentration of 100 μM (Figure 3C, Table 1). Lamivudine and zidovudine are nucleoside analogs that act as reverse transcriptase inhibitors, and they have been used to inhibit HIV infection [28]. However, neither drug reduced MERS-CoV RdRp activity at concentrations of up to 100 μM (Figure 3D,E, Table 1). We also tested the nucleoside analogs and viral DNA polymerase inhibitors valacyclovir and vidarabine, which inhibit HSV infection [29]. Neither drug inhibited MERS-CoV RdRp activity at concentrations of up to 100 μM (Figure 3F,G, Table 1). Especially vidarabine did not exert cytotoxicity (Figure S2H); however, concentration-dependent decreases of both FLuc and NLuc activity were unpredictably observed. This finding suggests that vidarabine inhibits host transcription/translation processes, but vidarabine had no effect on the NLuc/FLuc ratio or MERS-CoV RdRp activity.

### 3.4. The Non-Nucleoside HCV RdRp Inhibitor Dasabuvir Partially Inhibits MERS-CoV RdRp Activity

**Figure 4:**
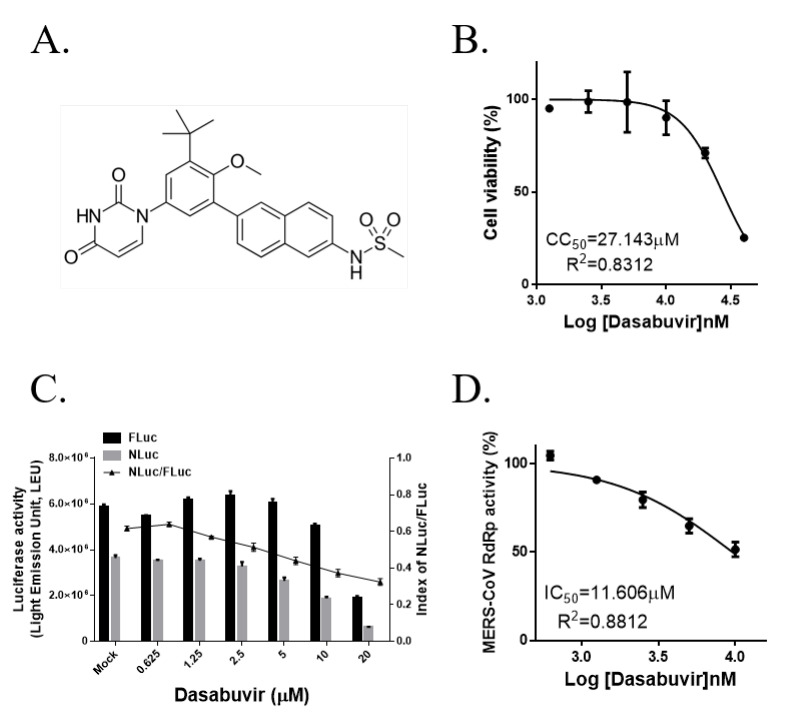
Effects of the non-nucleoside analog dasabuvir on Middle East respiratory syndrome coronavirus RNA-dependent RNA polymerase (RdRp) activity in the cell-based reporter assay system. (**A**) Chemical structure of dasabuvir. (**B**) The half-maximal cytotoxic concentration of dasabuvir was detected using 3-(4,5-dimethylthiazol-2-yl)-5-(3-carboxymethoxyphenyl)-2-(4-sulfophenyl)-2H-tetrazolium (MTS) assay after treatment in HEK293T cells for 18 h. (**C**) HEK293T cells were transiently transfected with *p*(+)FLuc-(−)UTR-NLuc and pN-termFlag-nsp12, and, after 6 h, cells were treated with the indicated concentrations of dasabuvir for 18 h. FLuc and NLuc activities were measured and the NLuc/FLuc ratio were determined. (**D**) The IC_50_ value of dasabuvir was calculated via non-linear regression analysis. The data, which are representative of at least three independent experiments, are presented as the mean ± SEM.

Dasabuvir is a derivative of benzothiadiazine (Figure 4A) that functions as a non-nucleoside inhibitor of HCV NS5B [13] by interacting with the conserved amino acids localized near the active site of the HCV NS5B palm domain [30]. We found that 10 μM dasabuvir reduced MERS-CoV RdRp activity by approximately 50% without any cytotoxicity and the IC_50_ was 11.606 μM according to non-linear regression analysis (Figure 4C,D), whereas cytotoxicity and reduced firefly luciferase activity were observed at concentrations exceeding 10 μM and the half-maximal cytotoxic concentration of dasabuvir was 27.143 μM in HEK293T cells (Figure 4B). Therefore, we could not detect the complete inhibition of MERS-CoV RdRp activity by dasabuvir.

### 3.5. Remdesivir (GS-5734) Inhibits MERS-CoV RdRp Activity in a Cell-Based Reporter Assay

**Figure 5:**
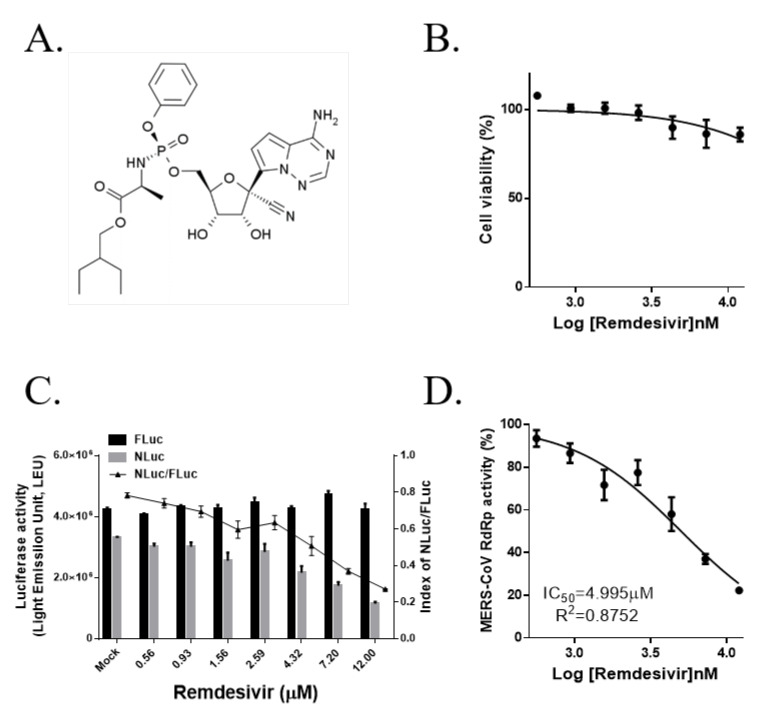
Effects of remdesivir on Middle East respiratory syndrome coronavirus RNA-dependent RNA polymerase activity in the cell-based reporter assay system. (**A**) Chemical structure of remdesivir. (**B**) The viability of remdesivir-treated HEK293T cells was determined using the MTS assay after treatment with the indicated concentrations for 18 h. (**C**) HEK293T cells were transiently transfected with p(+)FLuc-(−)UTR-NLuc and pN-termFlag-nsp12, and, after 6 h, cells were treated with remdesivir at the indicated concentrations for 18 h. FLuc and NLuc activities were measured and the NLuc/FLuc ratio were determined. (**D**) The IC_50_ value of remdesivir was calculated via non-linear regression analysis. The data, which are representative of at least three independent experiments, are presented as the mean ± SEM.

Remdesivir is a monophosphoramidate prodrug of an adenosine analog (Figure 5A). In prior research, remdesivir inhibited MERS-CoV infection in vitro with an IC_50_ of 0.074 ± 0.023 μM in human airway epithelial (HAE) cells, and remdesivir targets RdRp and exoribonuclease (nsp14, ExoN) in MHV, suggesting resistance mutation [5]. Therefore, we evaluated the direct inhibitory effects of remdesivir on MERS-CoV RdRp activity. The drug was demonstrated to reduce the relative NLuc activity in a dose-dependent manner while maintaining consistent FLuc activity, and no cytotoxicity was observed at concentrations of up to 12μM (Figure 5B,C). The IC_50_ of remdesivir was 5.028 ± 0.035 μM according to non-linear regression analysis (Figure 5D). There is the discrepancy between remdesivir IC_50_ of 0.074 ± 0.023 μM in MERS-CoV infected HAE cells and IC_50_ of 5.028 ± 0.035 μM in this system, which may be due to the different experimental conditions, using the artificially over-expressed MERS-CoV RdRp compared with using the infectious virus.

### 3.6. Reliability and Reproducibility of the Cell-Based MERS-CoV RdRp Activity Reporter Assay System in HTS

**Figure 6:**
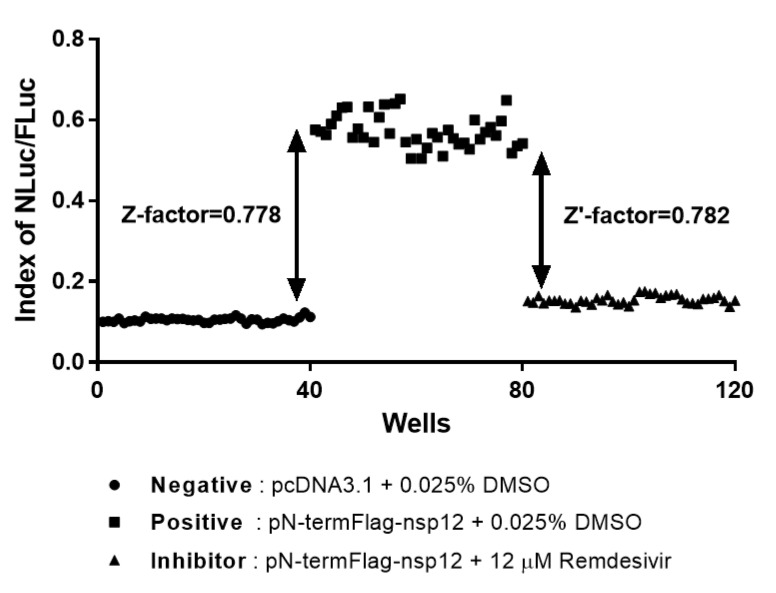
Validation of the accuracy of the cell-based reporter assay for Middle East respiratory syndrome coronavirus (MERS-CoV) RNA-dependent RNA polymerase (RdRp) as a high-throughput screening system. In the assay, 96-well plate-seeded HEK293T cells were transfected with p(+)FLuc-(−)UTR-NLuc and pcDNA3.1 followed by treatment with 0.025% DMSO (negative group, n = 40 wells), transfected with pN-termFlag-nsp12 followed by treatment with 0.025% DMSO (positive group, n = 40 wells), or transfected with pN-termFlag-nsp12 followed by treatment with 12 μM remdesivir (inhibitor group, n = 40 wells). Then, FLuc and NLuc activities were measured. According to Zhang’s formula, the Z-factor value between the positive and negative groups was 0.778, indicating that this assay system is excellent for detecting MERS-CoV RdRp activity. The Z′-factor between the positive and inhibitor groups was 0.782, indicating that remdesivir can be used as a positive control for MERS-CoV RdRp-specific inhibitors.

The Z-factor is the most widely used parameter in the evaluation and validation of HTS experiments [31]. In the present study, Z-factor was calculated using the relative NLuc activity obtained from the negative and positive groups to evaluate the discriminant ability of the assay for MERS-CoV RdRp activity. In addition, Z′-factor was calculated using data obtained from the positive and inhibitor groups to evaluate the applicability of remdesivir as a positive control for MERS-CoV RdRp inhibition. We obtained Z-factor and Z′-factor values of 0.778 and 0.782, respectively, indicating that the cell-based MERS-CoV RdRp activity reporter assay system reliably and reproducibly identifies MERS-CoV RdRp inhibitors in HTS systems (Figure 6).

## 4. Discussion

RdRp is one of the most important viral proteins of RNA viruses for RNA synthesis, and it has been suggested as a valuable target for the development of antiviral therapeutics. In the present study, we established a cell-based MERS-CoV RdRp activity reporter assay system by modifying the previously reported cell-based HCV RdRp activity assay [16]. This system consists of the bicistronic MERS-CoV RdRp reporter construct p(+)FLuc-(−)UTR-NLuc and pN-termFlag-nsp12 plasmid. The expression level of FLuc serves as an internal control, and the expression level of NLuc represents the activity of MERS-CoV RdRp. Because the N-term of RdRp is known to be important for protein folding and the positioning of the active site [18,19], we compared MERS-CoV RdRp activity between N-term and C-term FLAG-tagged RdRp, finding that the N-term FLAG tag did not interrupt the activity of MERS-CoV RdRp. We also found that the MERS-CoV 5′-UTR did not need the HCV IRES sequence for second cistron protein translation. Therefore, we finally selected p(+)FLuc-(−)UTR-NLuc without the HCV IRES element and pN-termFlag-nsp12 for the present assay system.

To assess RdRp enzyme activity using the purified recombinant RdRp protein, RNA template, RNA primer, and isotopic NTP are prepared for reaction, and radioisotope-labeling RNA products synthesized in a test tube are usually measured. In this case, there may be technical barriers that restrict the purification of a highly pure and bioactive RdRp protein, optimization of the RNA synthesis reaction conditions, and treatment using radioactive materials with guaranteed biosafety. However, the cell-based reporter assay system can overcome these difficulties, and the FLuc expression level, which serves as an internal control, directly indicates the cytotoxicity of test compounds, as observed for dasabuvir. Particularly, this system can determine whether the test compound affects host transcription/translation processes or specifically inhibits MERS-CoV RdRp activity. Moreover, we can screen the prodrug form of candidates, such as remdesivir, without the conversion process to obtain the pharmacologically active form because this system uses human cells.

SARS-CoV RdRp is known to be primer-dependent [32] and to need the interacting proteins, nsp8 and nsp7, in enzyme activity assay because the CoV nsp8 subunit that forms a hexadecameric complex with nsp7 functions as a RNA primase [20,21]. We also tested if nsp8 and nsp7 acts as the co-factors of RdRp’s gene synthesis. However, the nsp8 and nsp7 proteins did not give any significant effect on the activity of MERS-CoV RdRp in this cell-based system. Although we could not detect the expression of nsp7 protein in our system, it is consistent that when the MERS-CoV nsp12, nsp7, and nsp8 were co-expressed in insect cell system with nsp5 as a polyprotein, cleaved by the nsp5 protease, only nsp8 and nsp12, but not nsp7 were detected by SDS PAGE analysis and Mass spectroscopy [15]. Results were also published which found that SARS-CoV nsp12 purified in E.coli has primer–independent, de novo RNA synthesis activity without nsp7 and nsp8 proteins using viral RNA template containing the 3’ UTR of +/- strands of the SARS-CoV [33]. The mechanisms of RNA synthesis of coronaviruses have not well characterized in cell biology, so further studies are needed in more detail.

Using this system, we tested nucleoside/nucleotide analogs such as ribavirin, sofosbuvir, favipiravir, lamivudine, zidovudine, valacyclovir, vidarabine, and remdesivir, as well as the non-nucleoside analog dasabuvir, because they commonly target viral DNA or RNA polymerase [8]. Among them, the reverse transcriptase inhibitors lamivudine and zidovudine and DNA polymerase inhibitors valacyclovir and vidarabine did not significantly inhibit MERS-CoV RdRp activity. Meanwhile, favipiravir is known to selectively inhibit the PA protein of the influenza virus polymerase, which consists of three viral proteins (PA, PB1, and PB2), and induce lethal RNA transversion mutations, thereby producing a non-viable viral phenotype [26]. Despite acting as an active inhibitor against positive-stranded RNA viral RdRp [27], favipiravir only had weak inhibitory effects on MERS-CoV RdRp activity in our assay.

Ribavirin is a guanosine analog that functions as a broad-spectrum antiviral, and the drug was reported to interact with host inosine monophosphate dehydrogenase, which prevents viral RNA synthesis by depleting cellular guanosine triphosphate [34]. The incorporation of ribavirin triphosphate by RdRp also results in lethal viral mutagenesis [35]. Although ribavirin inhibited MERS-CoV infection in Vero RML6 and LLC-MK2 cells [14], these findings have failed to translate into clinical benefits in patients with MERS-CoV [36]. Our results illustrated that ribavirin only partially inhibited MERS-CoV RdRp activity, which could explain its failure in clinical trials.

Sofosbuvir is an uridine nucleotide analog that directly blocks the HCV NS5B protein (RdRp) and then inhibits RNA synthesis [25]. Our results indicated that this drug also partially inhibited MERS-CoV RdRp activity. We also examined the effect of the non-nucleoside HCV NS5B inhibitor dasabuvir [13], a derivative of benzothiadiazine, which interacts with the active site of the HCV NS5B palm domain [30]. The present assay illustrated that dasabuvir suppressed MERS-CoV RdRp activity at a low concentration, but full inhibition of the enzyme could not be observed because of toxicity at concentrations exceeding 10 μM. Although the sequence of viral RdRp in RNA viruses is versatile, the core structure of RdRp is conserved [37]. Moreover, alignment of the RdRp sequences between MERS-CoV and HCV revealed that the active site and its backbone site are conserved, suggesting that HCV RdRp inhibitors act also MERS-CoV RdRp inhibitors in a similar manner and to a similar extent [38]. However, ribavirin and sofosbuvir only partially inhibited MERS-CoV RdRp activity, whereas dasabuvir had stronger effects. These data indicate that HCV RdRp inhibitors do not universally inhibit MERS-CoV RdRp activity despite conservation of the active site between the viruses.

Remdesivir is a novel adenosine analog developed for treating Ebola virus infection as a chain terminator of viral RdRp [39], and it is a broad-spectrum antiviral drug with activity against RNA viruses including MHV, SARS-CoV, and MERS-CoV [5]. The target of remdesivir in coronaviruses was previously suggested to be the viral polymerase and ExoN of MHV based on the presence of remdesivir resistance mutations in the RdRp of MHV and an MHV mutant lacking ExoN, which is more susceptible to the drug [5]. Although remdesivir widely inhibits infection by coronaviruses with divergent RdRp sequences [40], this is the first study to demonstrate that remdesivir acts as a direct inhibitor of MERS-CoV RdRp activity expressed in the human cells based on its dose-dependent effects in a cell-based reporter assay.

We quantified the accuracy of this assay system by calculating Z-factor and Z′-factor, which are the most widely used parameters for evaluating and validating HTS experiments [31]. The present data indicated that this system is excellent for screening inhibitors of MERS-CoV RdRp activity. To avoid transient transfection experiment, we will generate the stable cell line harboring the reporter construct for the convenience of HTS experiments

## 5. Conclusions

We established a cell-based reporter assay for MERS-CoV RdRp activity to test viral polymerase inhibitors. Of the tested inhibitors, the cell-based reporter assay for MERS-CoV RdRp activity confirmed remdesivir as a direct inhibitor of MERS-CoV RdRp, and we clarified that this system is an accurate and useful HTS tool for screening specific and effective MERS-CoV RdRp inhibitors. Therefore, this system may provide a valuable platform for the development of effective antiviral therapeutics against MERS-CoV infection.

## Figures and Tables

**Table 1:** Percent activity of Middle East respiratory syndrome coronavirus (MERS-CoV) RNA-dependent RNA polymerase (RdRp) at the maximum concentrations of the test compounds.

Compound Name	Max Dose (μM)	MERS-CoV RdRp Activity (%)
Remdesivir	12	22.3 ± 0.3
Dasabuvir	10	51.5 ± 4.2
Ribavirin	100	60.3 ± 2.9
Sofosbuvir	100	62.4 ± 5.3
Favipiravir	100	85.8 ± 2.4
Lamivudine	100	98.3 ± 1.2
Zidovudine	100	110.8 ± 3.7
Vidarabine	100	89.9 ± 2.9
Valacyclovir	100	90.2 ± 3.2

## Supplementary Materials

The following are available online at https://www.mdpi.com/2077-0383/9/8/2399/s1, Figure S1: Relative Nano-glo^®^ luciferase (NLuc) activities by the nsp5 and nsp12 proteins, Figure S2: The viability of compound-treated HEK293T cells.

## Abbreviations

3CLpro	3C-like protease
CoV	coronavirus
COVID-19	coronavirus disease 2019
C-term	C-terminal
FLuc	firefly luciferase
HAE	human airway epithelial
HCV	hepatitis C virus
HDV	hepatitis delta virus
HIV	human immunodeficiency virus
HSV	herpes simplex virus
HTS	high-throughput screening
IRES	internal ribosome entry site
MERS	Middle East respiratory syndrome
MHV	mouse hepatitis virus
NLuc	Nano-glo^®^ luciferase
NSP	nonstructural proteins
N-term	N-terminal
PLpro	papain-like protease
RdRp	RNA-dependent RNA polymerase
SARI	severe acute respiratory infection
SARS	severe acute respiratory syndrome
UTR	untranslated region
