# Supplementary material for: A Cell-Based Reporter Assay for Screening Inhibitors of MERS Coronavirus RNA-Dependent RNA Polymerase Activity
Source: J Clin Med. 2020 Jul 27;9(8):2399. doi: 10.3390/jcm9082399 (PMC7465106; doi:10.3390/jcm9082399)
Supplement: Supplementary file 1 [file jcm-09-02399-s001.pdf]

Supplementary Figures

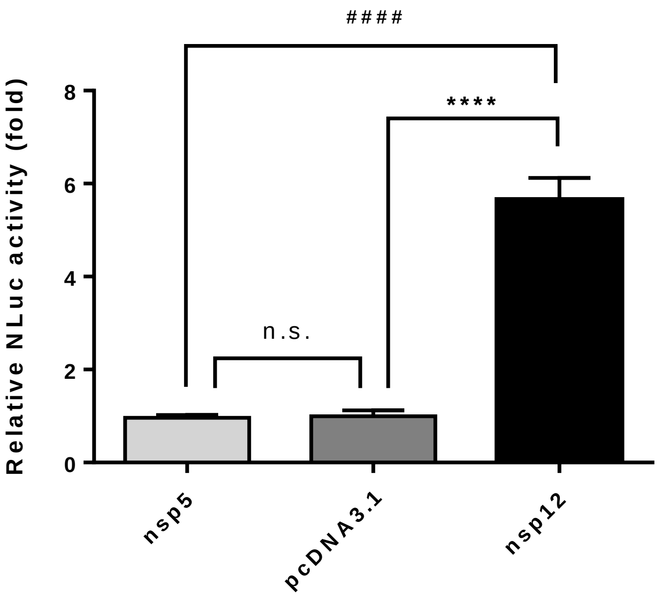

Figure S1. Relative Nano-glo® luciferase (NLuc) activities by the nsp5 and nsp12 proteins. HEK293T cells were transfected with nsp5 plasmids, pcDNA3.1 or nsp12 plasmids together with p(+)FLuc-(−)UTR-NLuc. After 24 h, FLuc and NLuc activities were detected using the Nano-glo® Dual-Luciferase® Reporter Assay System, and the relative NLuc activity was normalized by the FLuc activity. Data are presented as the mean ± SEM. Statistical significance was analyzed using one-way analysis of variance (ANOVA) followed by Bonferroni's multiple comparison. n.s., no significant; **** $p < 0.0001$; #### $p < 0.0001$. (n = 3 each; $F_{2,6} = 88.35$, $p < 0.0001$).

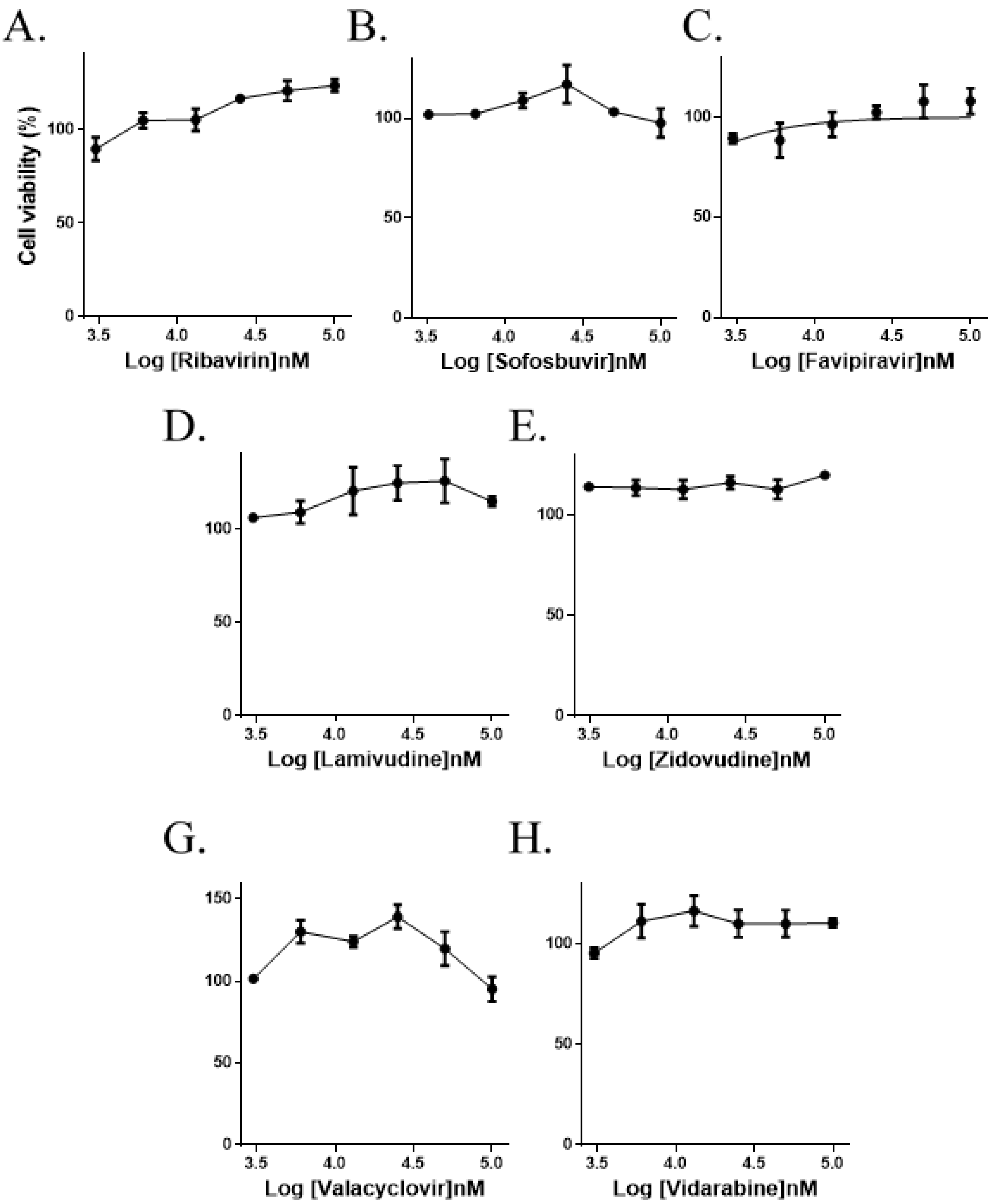

Figure S2. The viability of compound-treated HEK293T cells. HEK293T cells were treated with the indicated concentrations of ribavirin (A), sofosbuvir (B), favipiravir (C), lamivudine (D), zidovudine (E), valacyclovir (F), or vidarabine (G) for 18 h. Cell viability was determined using the MTS assay. The data, which are representative of at least three independent experiments, are presented as the mean ± SEM.
